# Prevalence and Trends in Low Bone Density, Osteopenia and Osteoporosis in U.S. Adults With Non-Alcoholic Fatty Liver Disease, 2005–2014

**DOI:** 10.3389/fendo.2021.825448

**Published:** 2022-01-19

**Authors:** Tianyu Zhai, Qi Chen, Jing Xu, Xi Jia, Pu Xia

**Affiliations:** ^1^ Department of Endocrinology and Metabolism, Zhongshan Hospital, and Fudan Institute for Metabolic Diseases, Fudan University, Shanghai, China; ^2^ State Key Laboratory of Pharmaceutical Biotechnology, The University of Hong Kong, Hong Kong, Hong Kong SAR, China; ^3^ Department of Medicine, The University of Hong Kong, Hong Kong, Hong Kong SAR, China

**Keywords:** non-alcoholic fatty liver disease, bone mineral density, osteopenia, osteoporosis, trend, spinal fracture, national health and nutrition examination survey

## Abstract

**Background & Aims:**

Non-alcoholic fatty liver disease (NAFLD) is suggested to be associated with bone mineral density (BMD) alterations; however, this has not been ascertained. The current study aimed to investigate the changes in BMD and the prevalence of osteopenia/osteoporosis in US adults with or without NAFLD and to evaluate their association.

**Methods:**

The study was conducted based on data collected from the U.S. National Health and Nutrition Examination Survey (NHANES) during the period 2005–2014. A total of 13 837 and 6 177 participants aged > 20 years were eligible for conducting the Hepatic Steatosis Index (HSI) and the US Fatty Liver Index (USFLI) analysis, respectively.

**Results:**

From 2005–2014, a downward trend in femoral neck BMD was observed in subjects with NAFLD aged ≥ 40. After adjustment for potential confounders, an upward shift occurred in the prevalence of osteopenia/osteoporosis at the femoral neck in adults aged ≥ 40, particularly in women ≥ 60 years old and men below the age of 60. Moreover, a negative association was found between BMD and NAFLD markers (USFLI, HSI), whereas NAFLD with advanced fibrosis was positively associated with the prevalence of spine fractures.

**Conclusions:**

There was a trend toward lower BMD and higher prevalence of osteopenia/osteoporosis at the femoral neck in US adults with NAFLD aged ≥ 40 years during the period of 2005–2014. NAFLD with advanced fibrosis was positively associated with a higher risk of spine fracture. More research is required to fully investigate the mechanism and consequence of poor bone health in NAFLD patients and consider optimum management of osteopenia/osteoporosis for this population.

## Introduction

Osteoporosis is a systemic skeletal disease characterized by low bone mass and deterioration of architecture, associated with excessive bone fragility and fracture risk (1). It is estimated that ~13 million individuals >50 years old are expected to suffer from osteoporosis by 2020 in US (2). During the period 1988–1994 to 2005–2006, a decline in the prevalence of osteoporosis was observed in the US population aged ≥ 50 years (3). According to the US Medicare data, a decrease in the rate of hip fractures was observed from 2002–2012, which plateaued during the period of 2013–2015 (4). In addition, by directly assessing the bone mineral density (BMD) data at various skeletal sites, more recent studies have confirmed a decline in femoral neck BMD in the US population from 2013–2014 (5).

Non-alcoholic fatty liver disease (NAFLD) has emerged as an noninfectious epidemic that threatens the health of ~24% in the global population, paralleling the increase of other metabolic conditions, including type 2 diabetes (T2DM), obesity, hyperlipidemia, hypertension, and metabolic syndrome (6). Osteoporosis accounts for most cases of hepatic osteodystrophy, which is defined as a skeletal disorder of multifactorial origin caused by chronic liver disease (7). As a chronic liver disease, NAFLD and the advanced histological phenotype non-alcoholic steatohepatitis (NASH), may have a potential link with osteoporosis, which has piqued considerable scientific interest (8).

Several cross-sectional studies have attempted to elucidate the association between NAFLD and BMD, osteoporosis, and relevant fractures (9–17). Not surprisingly, there are many conflicting findings in these studies, given that various factors including age, gender, race, and the nutritional and menstrual status may affect bone metabolism. Some studies have identified a significant association of lower BMD or higher rates of osteoporotic fracture with NAFLD in both adolescents and adults (9–13, 15), whereas other studies reported no significant associations and even opposite results (14, 16, 17). In addition, two recent meta-analyses also observed no significant association between fatty liver disease and decreased BMD (18, 19). Moreover, there is no evidence showing temporal trends of BMD and prevalence of osteopenia/osteoporosis in the individuals with NAFLD. Therefore, in the present study, by accessing the nationally representative data from the US National Health and Nutrition Examination Survey (NHANES) in the period of 2005–2014, we aimed to evaluate the temporal trends of BMD, osteopenia/osteoporosis in the US adults with or without NAFLD, and their possible associations.

## Methods

### Study Population

The cross-sectional analysis was conducted based on the 2005–2014 period data of NHANES that includes the multistage, stratified, clustered probability samples and represent the noninstitutionalized US population. NHANES obtained written informed consent from all individuals, which was approved by the National Center for Health Statistics institutional review board. The socioeconomic, demographic, medical, and dietary data were collected through health-related interviews, laboratory and physical evaluations.

### Study Design

**Figure 1:**
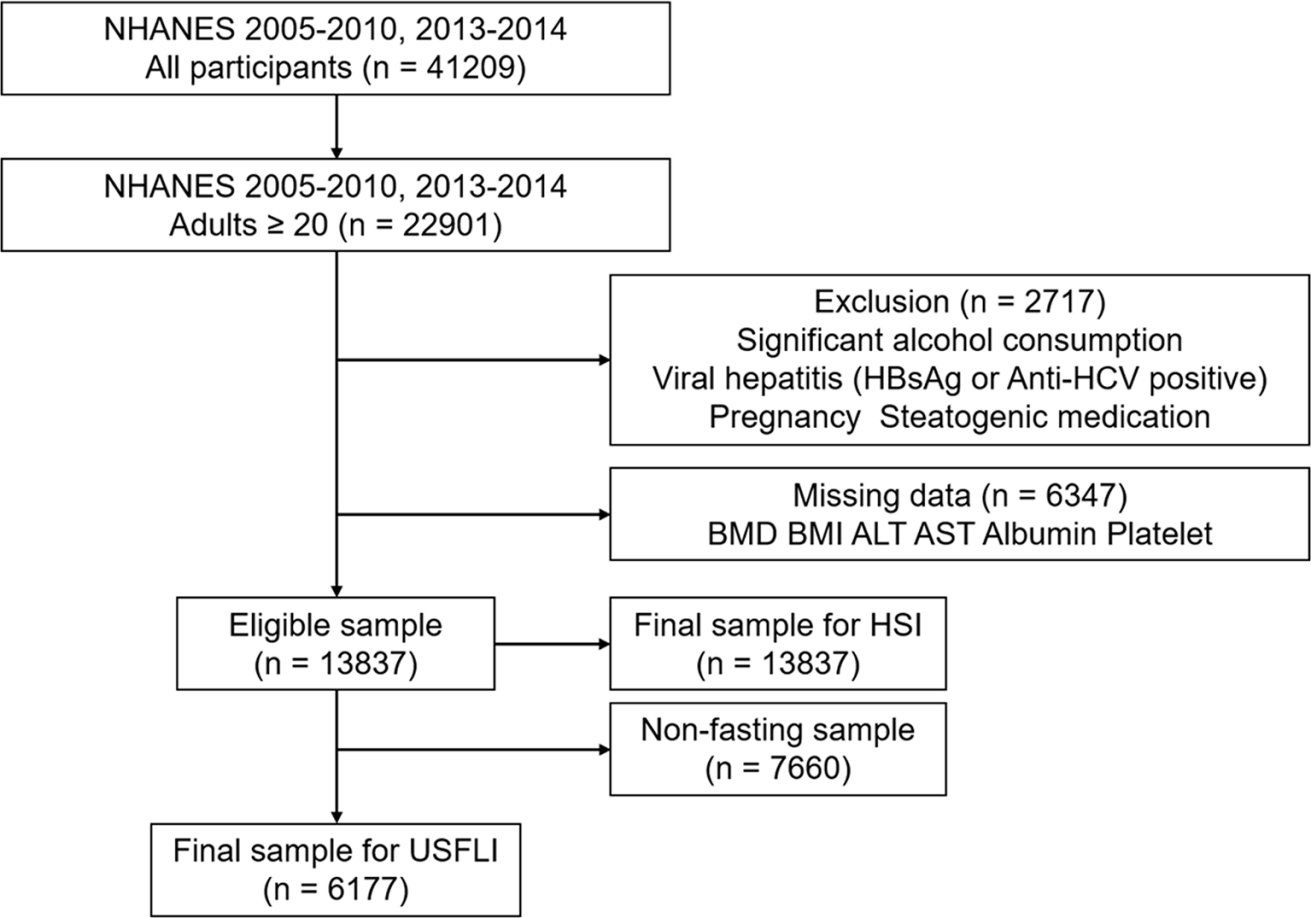
STROBE (STrengthening the Reporting of OBservational studies in Epidemiology) diagram of study participants.

Of all 41,209 participants in the 2005–2010 and 2013–2014 period in National Health and Nutrition Examination Survey (NHANES) 22 901 (55.9%) participants were > 20 years old. We excluded the participants with significant alcohol consumption (> 2 drinks/day for men or > 1 drink/day for women, n = 1,451), viral hepatitis (positive serum hepatitis B surface antigen and positive serum hepatitis C antibody, n = 421), pregnancy (n = 507), steatogenic medication (methotrexate, corticosteroids, valproate, amiodarone, and tamoxifen, n = 338) > 6 months, and individuals with missing data (BMD, Body mass index [BMI], serum aminotransferase, platelet count, n = 6,347). Additionally, 6,273 participants were lacking data regarding fasting blood glucose tests. Hence, the final cohort comprised 13,837 participants included in the subsequent analysis with Hepatic Steatosis Index (HSI), and 6,177 participants evaluated using the US Fatty Liver Index (USFLI) (**Figure 1**).

The data of BMD measurement of the proximal femur and lumbar spine are available from 2005–2014 except for 2011–2012. Additionally, only the participants aged ≥ 40 years have the BMD data for the 2013–2014 period. To reduce the variance of the estimates, the ages are categorized as 20–39, 40–59, and ≥ 60 years old. Therefore, we use the age of 40 as the limit in the main analysis and use ages 40 and 60 in the subgroup analysis stratified by age and sex. In the women aged ≥ 40 years, there are 1522 (64.6%) and 3373 (63.8%) postmenopausal women in USFLI and HSI analysis, respectively.

### Clinical and Laboratory Characteristics

The data of each participant was collected using household interviews, physical measurements, and laboratory tests in mobile examination centers (MECs). The information of age, sex, race/ethnicity, education level, economic status, milk intake, and history of fracture were collected using a standardized questionnaire. Race/ethnicity included Hispanic (Mexican American, other-Hispanic), non-Hispanic white and black, and other. Education level was based on whether the individual graduated from high school. Marital status was defined as married or cohabiting with a partner versus other. We defined current smokers as individuals who have smoked ≥ 100 cigarettes in their lifetime and are still cigarette users. We defined individuals with hypertension as having systolic blood pressure (SBP) > 140 mmHg, or diastolic blood pressure (DBP) > 90 mmHg and/or currently taking antihypertensive drugs. We defined the individuals with diabetes mellitus as having fasting plasma glucose (FPG) ≥ 126 mg/dL and/or currently receiving insulin or other hypoglycemic agent treatment. The family income-to-poverty ratio was used to evaluate the individual’s socioeconomic status: ≥ 1.00 = poverty, < 1.00 = below poverty level. Homoeostasis model assessment of insulin resistance (HOMA‐IR) was used to reflect insulin resistance (IR) (20). Milk intake was defined as never, rarely (< once a week), sometimes (< once a day and > once a week), often (> once a day), and varied. BMI was calculated as weight (kg) divided by the square of height (m). The metabolic equivalent (MET) data were collected using the Global Physical Activity Questionnaire from 2007–2008 and was used to evaluate the energy expenditure of individuals in one week (21).

The radioimmunoassay (DiaSorin) method was used to measure total 25-hydroxyvitamin D [25(OH)D] in 2005–2006, and Centers for Disease Control and Prevention (CDC) changed this method to liquid chromatography-tandem mass spectrometric (LC-MS/MS) method during the 2007–2010 period. For comparison, the CDC used regression equations to transform the 25(OH)D concentrations derived from radioimmunoassay to LC-MS/MS equivalents (21). There were no changes to the laboratory method, laboratory equipment, or laboratory site for this component in the NHANES 2013–2014 cycle.

### Definition of NAFLD and Advanced Fibrosis

For the definition of NAFLD, previously validated methods, including US Fatty Liver Index (USFLI)and Hepatic Steatosis Index (HSI), were used in epidemiologic studies in the absence of other causes of chronic liver disease and exposure to steatogenic medication. We used the published cut-off values of ≥ 30 and 36 for USFLI and HSI, respectively, to define the presence of NAFLD (22). Advanced fibrosis was evaluated according to a previous study (23), i.e., NAFLD fibrosis score (NFS) > 0.676 (high probability), -1.455 ≤ NFS ≤ 0.676 (intermediate probability), and NFS<-1.455 (low probability). We used the high probability of NFS score to define advanced fibrosis. The equations for calculation of the aforementioned markers are outlined in **Supplementary Table 1**.

### BMD Measurement and Definition of Osteopenia/Osteoporosis

DXA was conducted for BMD testing, and the detailed examination protocol is described on the official website of NHANES (https://www.cdc.gov/nchs/nhanes/index.htm). Hologic QDR-4500A fan-beam densitometer and Hologic Discovery model A densitometer (Hologic, Inc., Bedford, MA, USA) were utilized to perform the femur and spine scans in 2005–2010 and 2013–2014, respectively. In the 2005–2010 period, the femur scans were analyzed with Hologic Discovery v12.4, and spine scans were analyzed with APEX v3.0. APEX v4.0 was used for the analysis of both femur and spine scans in 2013–2014. A cross-calibration procedure was conducted to standardize the newer system to the legacy system by the Hologic Service Team (21). After the assessment, no difference was found between mean BMD at five femur regions analyzed by Discovery v12.4 and APEX v4.0 (24).

Osteopenia was defined as -2.5 < T-score ≤ -1.0, and osteoporosis was defined as T-score ≤ -2.5. T-score was calculated using the formula: T-score= (BMD_repondent_−mean BMD_reference group_)/SD_reference group_. As recommended by the World Health Organization (25), non-Hispanic white females aged 20–29 from the NHANES III report were included as the reference group for the T-score calculation of the femoral neck (26), and the reference group for the lumbar spine was obtained from the Vital and Health Statistics from the CDC (27).

### Definition of NAFLD and Advanced Fibrosis

For the definition of NAFLD, previously validated methods, including US Fatty Liver Index (USFLI)and Hepatic Steatosis Index (HSI), were used in epidemiologic studies in the absence of other causes of chronic liver disease and exposure to steatogenic medication. We used the published cut-off values of ≥ 30 and 36 for USFLI and HSI, respectively, to define the presence of NAFLD (22, 28). Advanced fibrosis was evaluated according to a previous study (23), i.e., NAFLD fibrosis score (NFS) > 0.676 (high probability), -1.455 ≤ NFS ≤ 0.676 (intermediate probability), and NFS<-1.455 (low probability). We used the high probability of NFS score to define advanced fibrosis. The equations for calculation of the aforementioned markers are outlined in **Supplementary Table 1**.

### Statistical Analysis

NHANES adopted the complex sampling design and provided representative data for the civilian, noninstitutionalized US population, which requires appropriate sample weight to adjust for the potential bias of non-response and the unequal probability of inclusion in the survey. Based on the principle of using the smallest subsample weight, we adopted the mobile examination center (MEC) exam weight and the fasting subsample weight in the current analysis. We calculated frequencies (standard errors, SE) for categorical variables and the means ± SE for continuous variables. We investigated the temporal prevalence of osteopenia/osteoporosis through the 2005–2014 period stratified by sex and age as recommended by the Centers for Disease Control and Prevention (CDC). Test for trends was calculated through a linear regression model by including the midpoint of each survey cycle as a continuous variable. Relative SE > 0.30 indicated low precision of the estimate.

Using the NHANES cycle of 2005–2006 as reference, we calculated the odds ratios (ORs) of osteopenia/osteoporosis over time *via* logistic regression analysis. Model 1 was adjusted for the sociodemographic variables, including sex, age, race, BMI, waist circumference, smoking, educational, marital, and economic status. Model 2 was adjusted for the adjustments of model 1 in addition to nutritional status, 25(OH)D, and milk intake. Model 3 was adjusted for hypertension, diabetes, high-density lipoprotein cholesterol (HDL-C), triglyceride (TG), total cholesterol (TC), and low-density lipoprotein (LDL-C) in addition to model 2. Model 4 was adjusted for the adjustments of model 3 plus menopausal status. Model 5 was further adjusted for physical activity.

Further, we evaluated the bone quality difference between controls and individuals with NAFLD or that with advanced fibrosis. We calculated the prevalence of osteopenia/osteoporosis and fracture, and investigated the association between NAFLD (with advanced fibrosis) and the odds of osteopenia/osteoporosis and fracture using logistic regression after adjustment for the significant factors as described above. Additionally, the association between NAFLD (with advanced fibrosis) and BMD was assessed by multivariate regression analysis in the adjusted models as described above. The NALFD or fibrosis markers (USFLI, HSI, and NFS) were taken as the independent variables, and femoral neck or lumbar spine BMD taken as the dependent variables. All analyses were conducted using SPSS version 24.0 Complex Survey module (IBM Corporation, Armonk, NY, USA). *P*<0.05 was considered statistically significant.

## Results

### Characteristics of Participants With or Without NAFLD From the 2005–2014 Period

Defined by USFLI, participants aged > 40 years with NAFLD exhibited a significant increasing trend with BMI, waist circumference, HOMA-IR, 25(OH)D, prevalence of diabetes and poverty, and a significant decreasing trend in the levels of TC, TG, LDL-C, prevalence of current smoker and marriage across the four NHANES cycle. Additionally, some variables, including age, sex, race, education level, proportions of hypertension, HDL-C level, and physical activity remained comparable from 2005–2014. In the participants aged ≥ 40 years old without NAFLD, compared with participants with NAFLD, the proportion of men and prevalence of hypertension significantly increased, whereas BMI, HOMA-IR, diabetes, and marriage were stable from 2005–2014 (**Table 1**). There were generally similar trends when NAFLD was defined using HSI (**Supplementary Table 2**).

**Table 1:** Characteristics of participants aged ≥ 40 with or without NAFLD defined by USFLI through 2005-2014 period.

	No NAFLD					NAFLD				
	2005-2006	2007-2008	2009-2010	2013-2014	*P* for trend	2005-2006	2007-2008	2009-2010	2013-2014	*P* for trend
**NAFLD by USFLI**										
**N**	566	745	788	813		259	478	528	381	
**Age (years)**	56.42 ± 0.56	56.19 ± 0.50	56.35 ± 0.48	56.62 ± 0.49	0.629	57.09 ± 0.85	57.76 ± 0.62	58.75 ± 0.60	58.37 ± 0.73	0.136
**Male (%)**	40.7 (2.5)	39.3 (2.3)	42.5 (2.2)	47.0 (2.3)	0.003	64.9 (3.7)	61.1 (2.9)	59.2 (2.8)	57.9 (3.3)	0.068
**Race (%)**					0.125					0.200
**Mexican Amercian**	4.4 (1.5)	3.9 (0.5)	3.9 (0.5)	4.6 (0.6)		10.7 (1.5)	8.9 (1.0)	11.9 (1.1)	13.7 (1.6)	
**Other Hispanic**	2.0 (0.7)	3.9 (0.5)	3.5 (0.4)	4.5 (0.6)		2.8 (1.2)	5.0 (0.7)	5.5 (0.7)	5.6 (1.0)	
**Non-Hispanic white**	76.1 (1.8)	75.0 (1.7)	74.8 (1.6)	69.1 (1.7)		78.3 (2.6)	78.4 (2.0)	73.7 (2.0)	70.6 (2.4)	
**Non-Hispanic black**	11.4 (1.1)	12.4 (1.1)	11.2 (1.0)	12.4 (1.0)		3.9 (0.9)	3.7 (0.7)	4.4 (0.8)	5.4 (1.0)	
**Other races**	6.0 (1.2)	4.8 (1.1)	6.6 (1.2)	9.3 (1.0)		4.3 (1.7)	3.9 (1.4)	4.4 (1.3)	4.7(0.9)	
**BMI (Kg/m^2^)**	26.50 ± 0.23	26.48 ± 0.21	26.16 ± 0.20	26.77 ± 0.20	0.231	32.93 ± 0.40	32.12 ± 0.30	32.54 ± 0.30	33.31 ± 0.44	0.050
**Waist circumference (cm)**	93.08 ± 0.60	93.44 ± 0.52	92.68 ± 0.50	94.59 ± 0.50	0.008	111.58 ± 0.91	109.79 ± 0.65	110.58 ± 0.66	112.75 ± 0.98	0.017
**Current smoker (%)**	20.0 (2.1)	18.2 (1.8)	12.1 (1.4)	16.6 (1.7)	0.060	19.7 (3.1)	12.9 (2.1)	12.3 (1.7)	11.6 (2.2)	0.009
**Hypertension (%)**	30.9 (2.3)	35.5 (2.2)	32.5 (2.0)	37.3 (2.2)	0.027	56.2 (3.8)	55.3 (3.1)	55.6 (2.8)	50.8 (3.3)	0.121
**Diabetes (%)**	7.8 (1.1)	8.3 (1.0)	8.3 (1.1)	7.3 (0.9)	0.655	21.6 (3.1)	27.0 (2.5)	26.6 (2.4)	35.3 (3.2)	< 0.001
**Married status (%)**	73.3 (2.1)	68.1 (2.1)	72.9 (1.8)	72.6 (2.0)	0.611	73.3 (3.4)	78.1 (2.2)	74.1 (2.4)	66.8 (3.1)	0.003
**High education (%)**	84.4 (1.6)	84.1 (1.4)	86.1 (1.3)	86.5 (1.3)	0.156	80.6 (2.6)	78.5 (2.3)	72.8 (2.3)	75.9 (2.7)	0.106
**Poverty (%)**	7.7 (1.1)	10.3 (1.2)	8.0 (1.0)	12.0 (1.3)	0.016	7.7 (1.6)	8.9 (1.4)	11.3 (1.4)	12.3 (1.9)	0.034
**HOMA-IR**	1.69 ± 0.04	1.89 ± 0.04	2.00 ± 0.04	1.77 ± 0.04	0.513	5.57 ± 0.33	6.20 ± 0.32	6.32 ± 0.25	7.72 ± 0.86	0.002
**25(OH)D (nmol/L)**	63.74 ± 1.08	70.56 ± 1.18	75.19 ± 1.15	76.15 ± 1.33	<0.001	55.68 ± 1.27	64.82 ± 1.20	66.06 ± 1.46	68.16 ± 1.62	<0.001
**Total cholesterol (mg/dL)**	205.65 ± 1.91	204.39 ± 1.83	204.44 ± 1.94	196.78 ± 1.78	<0.001	204.76 ± 3.69	198.80 ± 2.60	195.05 ± 2.09	191.81 ± 2.87	<0.001
**Triglyceride (mg/dL)**	120.21 ± 3.48	117.22 ± 3.31	113.38 ± 4.11	103.53 ± 3.02	<0.001	211.65 ± 13.8	176.70 ± 6.57	164.26 ± 5.24	161.32 ± 6.05	<0.001
**HDL-cholesterol (mg/dL)**	59.57 ± 0.84	58.60 ± 0.76	60.03 ± 0.71	58.33 ± 0.78	0.256	46.13 ± 0.86	46.76 ± 0.60	46.06 ± 0.65	45.79 ± 0.70	0.398
**LDL-cholesterol (mg/dL)**	122.24 ± 1.74	122.45 ± 1.58	121.61 ± 1.62	117.81 ± 1.61	0.006	119.37 ± 3.31	117.75 ± 2.27	117.03 ± 1.78	113.47 ± 2.50	0.024
**MET (min/week)^1^**	–	4233 ± 310	3430 ± 211	3617 ± 253	0.121	–	3716 ± 340	3724 ± 367	3183 ± 310	0.162
**Menopause (%)^2^**	62.4 (3.5)	60.4 (3.0)	55.2 (2.9)	66.9 (2.8)	0.156	72.5 (5.9)	76.4 (4.1)	70.7 (3.9)	77.0 (3.7)	0.536
**Milk intake (%)**					0.005					0.001
**Never**	15.7 (1.9)	19.4 (1.8)	16.4 (1.7)	19.4 (1.8)		12.7 (2.7)	12.7 (2.1)	15.3 (2.1)	19.2 (2.8)	
**Rarely**	11.5 (1.7)	14.0 (1.7)	15.0 (1.6)	15.4 (1.6)		16.3 (2.8)	14.4 (2.0)	9.6 (1.5)	15.3 (2.4)	
**Sometimes**	24.2 (2.2)	26.3 (2.1)	23.0 (1.9)	26.0 (2.0)		26.1 (3.4)	29.7 (2.8)	32.0 (2.7)	30.5(3.0)	
**Often**	48.2 (2.6)	39.4 (2.3)	45.4 (2.2)	39.0 (2.2)		44.0 (3.8)	43.0 (3.1)	42.9 (2.8)	35.0 (3.2)	
**Varied**	0.4 (0.3)	0.8 (0.4)	0.2 (0.2)	0.3 (0.1)		1.0 (0.9)	0.2 (0.2)	0.1 (0.1)	0.2 (0.4)	
**BMD (g/cm^2^)**										
**Femoral neck**	0.78 ± 0.007	0.79 ± 0.006	0.79 ± 0.006	0.77 ± 0.006	0.070	0.84 ± 0.010	0.85 ± 0.008	0.82 ± 0.007	0.80 ± 0.011	<0.001
**Lumbar spine**	1.02 ± 0.009	1.01 ± 0.008	1.02 ± 0.008	1.00 ± 0.008	0.140	1.06 ± 0.012	1.04 ± 0.010	1.05 ± 0.011	1.04 ± 0.016	0.151

Data are expressed as mean ± SE for continuous variables or the proportion (SE) for categorical variables. ^1^MET score calculation was not available in 2005-2006 period. ^2^The proportion of Menopause was calculated in women.

Remarkably, a significant decreasing trend of BMD was observed at the femoral neck in participants with NAFLD aged ≥ 40 years old throughout the decade. The BMD at the lumbar spine did not change, nor there was a trend. These findings were consistent when NAFLD was defined using HSI (**Table 1** and **Supplementary Table 2**).

Besides, in the 20–39 age group of participants with or without NAFLD, significantly decreasing trends of marriage and amount of physical activity were observed from 2005–2014. However, the trend concerning femoral neck and lumbar spine BMD remained stable across the three cycles (**Supplementary Table 3**).

### Bone Status of Participants With or Without NAFLD Stratified by Age and Sex

**Figure 2:**
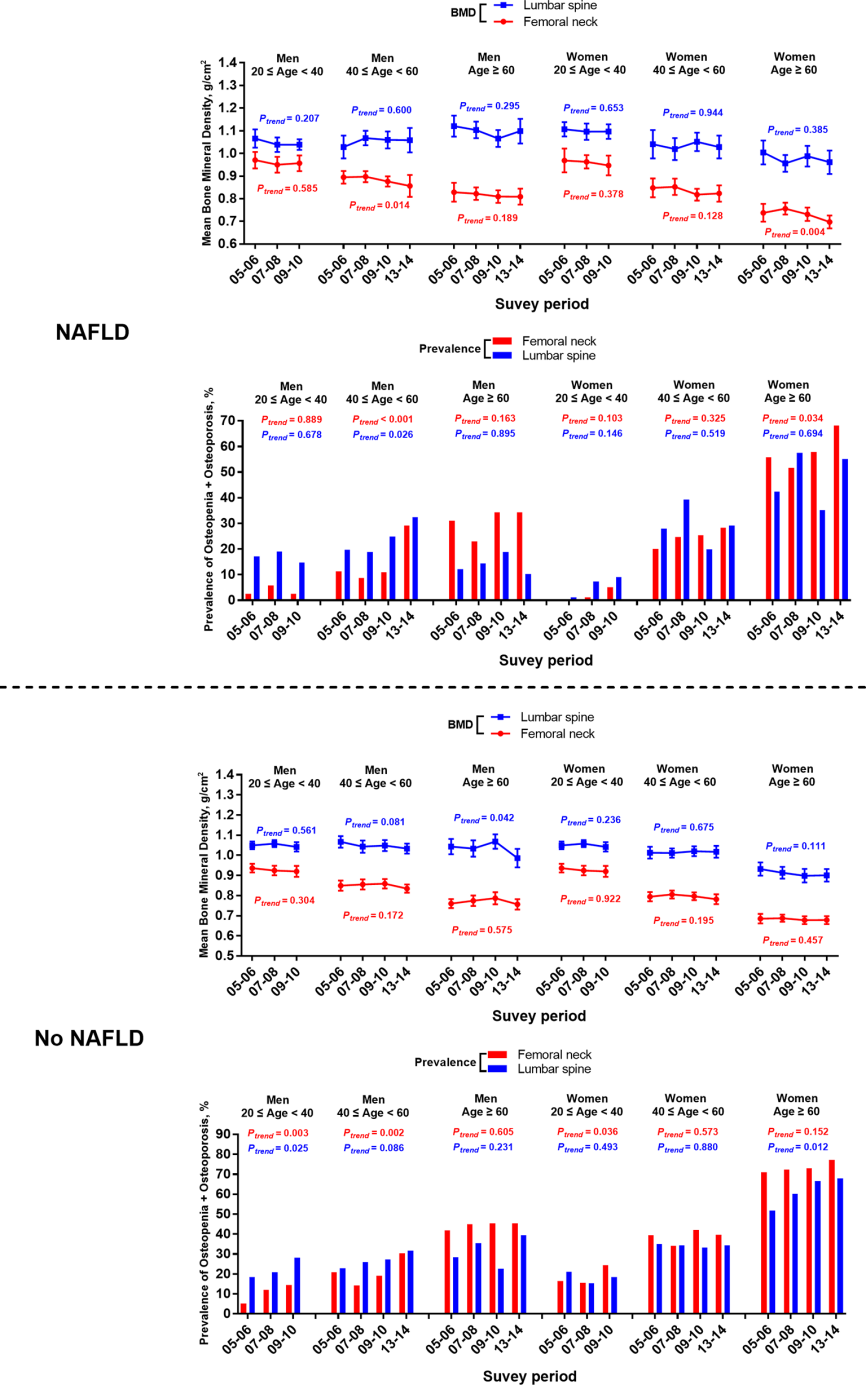
Temporal changes in BMD and prevalence of osteopenia/osteoporosis at femoral neck and lumbar spine in participants with or without NAFLD defined by USFLI, stratified by age and sex from 2005-2014. BMD is expressed as mean and 95% CI.

Throughout the survey cycles, a significant declining trend of femoral neck BMD was observed in men aged 40–59 years old and women aged > 60 with NAFLD defined by both USFLI and HSI. Accordingly, the prevalence of osteopenia and osteoporosis at the femoral neck showed an increasing trend in the two groups (**Figure 2** and **Supplementary Figure 1**). Additionally, in the analysis of participants with NAFLD defined by HSI, we observed a significant declining trend of BMD and an increasing trend in the prevalence of osteopenia and osteoporosis at the femoral neck in men aged > 60 years old and women aged 40–59 across the cycles.

Among the participants without NAFLD from 2005–2014, there was a significantly elevated trend of the prevalence of femoral neck osteopenia and osteoporosis in men aged 40-59 defined by either USFLI or HSI (**Figure 2** and **Supplementary Figure 1**). Meanwhile, women aged over 60 without NAFLD defined by USFLI had a significantly elevated trend of the prevalence of osteopenia and osteoporosis in lumbar spine through the decade (**Figure 2**).

Furthermore, in the subgroup of participants from 20–39 years old, men without NAFLD defined by the two markers showed significantly growing trends in the prevalence of osteopenia and osteoporosis in both sites from 2005–2010, and the rising trend of this index was also observed in the femoral neck in women without NAFLD defined by USFLI (**Figure 2** and **Supplementary Figure 1**).

### Association Between the Prevalence of Osteopenia/Osteoporosis and the Survey Cycle in the Participants With or Without NAFLD

In participants aged ≥ 40 years with and without NAFLD defined by USFLI, after adjustment for sociodemographic characteristics, BMI, waist circumstance, nutritional and menopausal status, NAFLD-related blood biochemical indices, and other metabolic disorders (model 4), a more recent survey cycle was positively associated with the prevalence of femoral neck osteopenia and osteoporosis (2013–2014 vs. 2005–2006; OR: 2.40, 95% CI: 1.38–4.19; *p* = 0.001 and OR: 1.59, 95% CI: 1.11–2.26; *p* = 0.004, respectively). This significance was not altered after further adjustment for physical activity (MET score) (model 5; *p* < 0.001 and p = 0.008, respectively) (**Table 2**). As for the lumbar spine, no significant cycle trend for the prevalence of osteopenia and osteoporosis was observed in the final adjusted model in participants aged ≥ 40 years with or without NAFLD in the fully adjusted model (model 4, 2013–2014 vs. 2005–2006 OR: 1.96, 95% CI: 1.02–3.78; *p* = 0.197 and OR: 1.50, 95% CI: 1.02–2.18, *p* = 0.212). In addition, adjustment for physical activity further rendered the cycle trend towards marginally insignificant in participants with NAFLD (model 4, *p* = 0.094) (**Table 2**).

**Table 2:** Trends in prevalence of osteopenia/osteoporosis in participants aged ≥ 40 with or without NAFLD defined by USFLI through 2005-2014 period.

NAFLD by USFLI	No NAFLD					NAFLD				
	2005-2006	2007-2008	2009-2010	2013-2014	*P* for trend	2005-2006	2007-2008	2009-2010	2013-2014	*P* for trend
**Femoral neck**										
** Prevalence (%)**	41.2	38.6	42.6	45.8		24.9	23.1	29.2	38.1	
** Model 1**	1.00 (Ref.)	0.77 (0.55-1.09)	1.01 (0.73-1.39)	1.37 (0.99-1.91)	0.009	1.00 (Ref.)	0.87 (0.52-1.45)	1.08 (0.65-1.78)	2.02 (1.19-3.42)	0.002
** Model 2**	1.00 (Ref.)	0.80 (0.56-1.15)	1.08 (0.78-1.51)	1.49 (1.06-2.10)	0.006	1.00 (Ref.)	1.01 (0.60-1.71)	1.20 (0.72-1.99)	2.33 (1.36-4.00)	0.001
** Model 3**	1.00 (Ref.)	0.84 (0.58-1.20)	1.14 (0.81-1.59)	1.60 (1.12-2.27)	0.003	1.00 (Ref.)	1.04 (0.61-1.75)	1.34 (0.81-2.22)	2.48 (1.44-4.27)	0.001
** Model 4**	1.00 (Ref.)	0.84 (0.58-1.21)	1.18 (0.84-1.66)	1.59 (1.11-2.26)	0.004	1.00 (Ref.)	0.98 (0.57-1.68)	1.37 (0.81-2.31)	2.40 (1.38-4.19)	0.001
** Model 5**	–	1.00 (Ref.)	1.52 (1.00-2.29)	1.93 (1.27-2.92)	0.008	–	1.00 (Ref.)	1.50 (0.85-2.66)	3.57 (1.92-6.64)	<0.001
**Lumbar spine**										
** Prevalence (%)**	33.5	36.1	35.3	39.1		23.4	29.5	24.1	32.6	
** Model 1**	1.00 (Ref.)	1.14 (0.80-1.62)	1.07 (0.74-1.53)	1.39 (0.97-1.99)	0.304	1.00 (Ref.)	1.27 (0.75-2.17)	0.96 (0.54-1.70)	1.55 (0.83-2.91)	0.321
** Model 2**	1.00 (Ref.)	1.15 (0.80-1.67)	1.09 (0.76-1.57)	1.41 (0.98-2.03)	0.293	1.00 (Ref.)	1.46 (0.84-2.55)	1.03 (0.58-1.84)	1.71 (0.91-3.25)	0.181
** Model 3**	1.00 (Ref.)	1.17 (0.81-1.71)	1.12 (0.77-1.63)	1.50 (1.03-2.18)	0.183	1.00 (Ref.)	1.56 (0.89-2.75)	1.16 (0.65-2.06)	1.98 (1.03-3.79)	0.138
** Model 4**	1.00 (Ref.)	1.18 (0.81-1.72)	1.16 (0.80-1.70)	1.50 (1.02-2.18)	0.212	1.00 (Ref.)	1.47 (0.83-2.60)	1.19 (0.66-2.11)	1.96 (1.02-3.78)	0.197
** Model 5**	-	1.00 (Ref.)	1.00 (0.65-1.54)	1.27 (0.81-1.97)	0.445	-	1.00 (Ref.)	0.82 (0.42-1.62)	1.78 (0.89-3.55)	0.094

Data are expressed as OR (95% CI). Model 1 was adjusted for sex, age, race, BMI, waist circumference smoking, educational, marital, and economic status. Model 2 was adjusted for the adjustments of model 1 plus nutritional status, 25(OH)D and milk intake included. Model 3 was adjusted for hypertension, diabetes, HDL-C, TG, TC, and LDL-C in addition to model 2. Model 4 was adjusted for the adjustments of model 3 plus menopausal status. Model 5 was further adjusted for physical activity, not available for 2005-2006 period.

Similar results were observed in the analysis when NAFLD was defined by HSI, as shown in **Supplementary Table 4**.

### The Association Between Bone Status and NAFLD or NAFLD With Advanced Fibrosis

Compared with controls, the femoral neck and lumbar spine BMD were significantly greater in participants with NAFLD or NAFLD with advanced fibrosis (**Supplementary Table 5**). In participants aged over 40, BMD in both sites exhibited a significantly negative association with USFLI in the final adjusted model (model 5, β = -0.0004, 95% CI: -0.0008 to -0.0001; *p* = 0.028, and β = -0.0006, 95% CI: -0.0011 to -0.0001; *p* = 0.033). Similar results were observed in the analysis when NAFLD was defined by HSI. A significantly negative association was also observed between NFS and BMD of the femoral neck, but not lumbar spine (β = -0.0070, 95% CI -0.0123 to -0.0015, *p* = 0.012, and β = 0.0046, 95% CI -0.0034 to 0.0126, *p* = 0.262) (**Table 3**).

**Table 3:** The correlation between NAFLD markers (USFLI, HSI, NFS) and BMD at femoral neck and lumbar spine in participants aged ≥ 40.

	Femoral neck BMD	*P*	Lumbar spine BMD	*P*
**USFLI**	***β* (95% CI)**		***β* (95% CI)**	
** Model 1**	-0.0005 (-0.0007, -0.0002)	<0.001	-0.0005 (-0.0008, -0.0001)	0.012
** Model 2**	-0.0005 (-0.0007, -0.0002)	<0.001	-0.0004 (-0.0008, -0.0001)	0.020
** Model 3**	-0.0004 (-0.0007, -0.0002)	0.002	-0.0005 (-0.0009, -0.0001)	0.021
** Model 4**	-0.0004 (-0.0007, -0.0002)	0.002	-0.0005 (-0.0009, -0.0001)	0.032
** Model 5**	-0.0004 (-0.0008, -0.0001)	0.028	-0.0006 (-0.0011, -0.0001)	0.033
**HSI**				
** Model 1**	-0.0022 (-0.0033, -0.0011)	<0.001	-0.0048 (-0.0064, -0.0032)	<0.001
** Model 2**	-0.0021 (-0.0033, -0.0010)	<0.001	-0.0049 (-0.0065, -0.0032)	<0.001
** Model 3**	-0.0027 (-0.0044, -0.0009)	0.004	-0.0050 (-0.0070, -0.0020)	<0.001
** Model 4**	-0.0021 (-0.0039, -0.0004)	0.019	-0.0037 (-0.0062, -0.0011)	0.005
** Model 5**	-0.0023 (-0.0047, 0.0001)	0.062	-0.0037 (-0.0070, -0.0003)	0.032
**NFS**				
** Model 1**	-0.0069 (-0.0092, -0.0046)	<0.001	0.0062 (0.0026, 0.0098)	<0.001
** Model 2**	-0.0067 (-0.0091, -0.0043)	<0.001	0.0063 (0.0027, 0.0099)	<0.001
** Model 3**	-0.0106 (-0.0146, -0.0067)	<0.001	0.0019 (-0.0041, 0.0079)	0.528
** Model 4**	-0.0100 (-0.0138, -0.0060)	<0.001	0.0015 (-0.0045, 0.0074)	0.629
** Model 5**	-0.0070 (-0.0123, -0.0015)	0.012	0.0046 (-0.0034, 0.0126)	0.262

Data are expressed as β coefficient (95% CI). Model 1 was adjusted for sex, age, race, BMI, waist circumference smoking, educational, marital, and economic status. Model 2 was adjusted for the adjustments of model 1 plus nutritional status, 25(OH)D and milk intake included. Model 3 was adjusted for hypertension, diabetes, HDL-C, TG, TC, and LDL-C in addition to model 2. Model 4 was adjusted for the adjustments of model 3 plus menopausal status. Model 5 was further adjusted for physical activity, not available for 2005-2006 period.

Besides, although no significant association was observed between NAFLD and hip or spine fracture in the participants aged ≥ 40 years (model 5, OR: 0.66, 95% CI: 0.12–3.61; *p* = 0.629, and OR 0.65, 95% CI 0.23–1.79; *p* = 0.400), the association was significantly positive between advanced fibrosis and spine fracture (model 5, OR: 3.75, 95% CI: 1.04–13.53; *p* = 0.044) (**Table 4**). Similar results were observed in the analysis when NAFLD was defined by HSI (**Supplementary Table 6**).

**Table 4:** The association between NAFLD (defined by USFLI) or NAFLD with advanced fibrosis and odds of fracture in participants aged ≥ 40.

NAFLD by USFLI	No NAFLD	NAFLD	*P*	No NAFLD with Advanced Fibrosis	NAFLD with Advanced Fibrosis	*P*
Hip fracture						
Prevalence (%)	1.4	1.4		1.3	2.3	
** Model 1**	1.00 (Ref.)	0.91 (0.45-1.82)	0.782	1.00 (Ref.)	1.38 (0.34-5.59)	0.650
** Model 2**	1.00 (Ref.)	0.88 (0.45-1.73)	0.715	1.00 (Ref.)	1.30 (0.31-5.40)	0.717
** Model 3**	1.00 (Ref.)	1.05 (0.47-2.33)	0.904	1.00 (Ref.)	1.10 (0.26-4.60)	0.900
** Model 4**	1.00 (Ref.)	1.05 (0.47-2.35)	0.903	1.00 (Ref.)	1.25 (0.30-5.28)	0.758
** Model 5**	1.00 (Ref.)	0.66 (0.12-3.61)	0.629	1.00 (Ref.)	3.34 (0.54-20.88)	0.196
Spine fracture						
Prevalence (%)	2.2	3.1		2.4	6.1	
** Model 1**	1.00 (Ref.)	0.91 (0.45-1.83)	0.796	1.00 (Ref.)	2.61 (0.81-8.42)	0.108
** Model 2**	1.00 (Ref.)	0.88 (0.43-1.79)	0.719	1.00 (Ref.)	2.65 (0.81-8.71)	0.108
** Model 3**	1.00 (Ref.)	0.74 (0.36-1.55)	0.426	1.00 (Ref.)	2.60 (0.84-8.08)	0.098
** Model 4**	1.00 (Ref.)	0.74 (0.35-1.54)	0.416	1.00 (Ref.)	2.57 (0.82-8.03)	0.105
** Model 5**	1.00 (Ref.)	0.65 (0.23-1.79)	0.400	1.00 (Ref.)	3.75 (1.04-13.53)	0.044

Data are expressed as OR (95% CI). Model 1 was adjusted for sex, age, race, BMI, waist circumference smoking, educational, marital, and economic status. Model 2 was adjusted for the adjustments of model 1 plus nutritional status, 25(OH)D and milk intake included. Model 3 was adjusted for hypertension, diabetes, HDL-C, TG, TC, and LDL-C in addition to model 2. Model 4 was adjusted for the adjustments of model 3 plus menopausal status. Model 5 was further adjusted for physical activity, not available for 2005-2006 period.

## Discussion

In the current study we have for the first time revealed a downward shift in the femoral neck BMD in the US adults ≥ 40 years with NAFLD based on NHANES data from the 2005–2006 to the 2013–2014 period. After adjustment for potential confounders, including sociodemographic characteristics, BMI, waist circumstance, nutritional and menopausal status, NAFLD-related blood biochemical indices, lifestyle-related factors, and other metabolic disorders, we observed an upward shift in the prevalence of osteopenia/osteoporosis at the femoral neck in adults aged ≥ 40 years over the period of 2005-2014, particularly in women > 60 years old and men below age 60. Additionally, an increasing trend in the prevalence of osteopenia/osteoporosis at both the femoral neck and lumbar spine was observed in men aged 20–39 years without NAFLD since 2005. Remarkably, we found a negative association between BMD at both sites and all NAFLD markers (USFLI, HSI and NFS). Moreover, NAFLD with advanced fibrosis was positively associated with the occurrence of spine fracture.

It has been reported that a downward trend of bone mass existed in older individuals in the US from 2005–2014 (24). Our results further uncovered a declining trend of BMD in subjects with NAFLD in the US. This could have a significant clinical implication, given the fact that the prevalence of NAFLD reached 31.1% of NHANES participants in the 2015–2016 period. It is noted that the trend of bone loss in the femoral neck appears more obvious than that in lumbar spine. The inconsistent trend at the two bone sites may be attributable to the different bone loss patterns (29). Another possible reason is that the measurement of lumbar spine BMD could be confounded by certain factors, including osteophytes and aortic calcification (30).

Apparently, in the subgroup analysis, there was a more significant downward trend of femoral neck BMD loss in men aged 40–59 years and women aged > 60 with NAFLD defined by USFLI. Accordingly, the two age groups had a growing trend of the prevalence of osteopenia and osteoporosis at the femoral neck. Hip fractures commonly cause more disability than fractures at other sites and are closely related to low BMD at the femoral neck (31). Indeed, existing evidence indicates that BMD at the femoral neck has the strongest ability to predict hip fracture (32). Another remarkable observation from the current study is that the prevalence of osteopenia/osteoporosis in younger men aged 20–39 is also increasing, not just in older adults. Nonetheless, there is the misconception that osteoporosis only occurs in the elderly or postmenopausal women. Currently, the majority of men at high risk of osteoporosis or fracture remain underappreciated and undertreated (33). Compared with women, there is a lack of long-term and powered studies of osteoporosis in men. Educational programs should be conducted not only in postmenopausal women but also in young and middle-aged men to prevent bone-related disorders, particularly in individuals with NAFLD.

In order to determine the possible factors that truly affect this upward shift of osteopenia/osteoporosis at the femoral neck, we investigated changes in sociodemographic characteristics, BMI, waist circumstance, nutritional status, NAFLD-related blood biochemical indices, lifestyle-related factors, and other metabolic disorders between survey cycles. After adjustment for the variables in model 4, we did not observe a marked alteration in the trend, which indicated that these variables perhaps do not play a primary role in the shift of bone status at the femoral neck. One important factor is the amount of physical activity, given that a sedentary lifestyle is associated with osteoporosis and decreased BMD(2004), and subjects with NAFLD are less likely to engage in physical activity and spend more sedentary time than controls. In our analysis, most notably, the MET scores decreased from 3716 and 3724 to 3183 min/week among individuals with NAFLD defined by USFLI and from 4583 to 4066 to 3645 min/week among those with NAFLD defined by HSI through the periods; adjustment for physical activity rendered the cycle trends of prevalence of lumbar spine osteopenia and osteoporosis into marginally insignificant in participants with NAFLD. Hence, the decreased MET scores across the survey periods may explain the decreasing BMD trend.

Over the past decades, there has been a growing awareness of the association between bone health and NAFLD. Nonetheless, previous studies have reported conflicting results that NALFD is associated with lower, similar, or higher BMD compared with healthy controls. Various factors may interfere with the findings, such as age (children, adolescent, adult), sex (male, female), race (Hispanic, Non-Hispanic, Asian, others), menstrual status, measurement technology of BMD, diagnosis of NAFLD, sample size and other unknown confounding factors. In our current analysis, compared with participants without NAFLD, we found a higher BMD and a lower prevalence of osteopenia/osteoporosis both at the femoral neck and lumbar spine in individuals with NAFLD. It is possible that BMI has the most significant and strongest effect on the BMD difference (18). The literature has revealed that BMD is positively associated with BMI (34), and greater BMD was observed in obesity, regardless of the bone site and measurement methodology (35). The excessive body fat mass and lean mass lead to increased passive loading, and muscle-related strain, so enhanced formation of cortical bone in obesity may help explain these findings (36). On the other hand, the chronic inflammation caused by adiposity might be detrimental to bone health. The overproduction of several proinflammatory cytokines, including interleukin (IL)-1 (IL-1) and IL-6, encourage bone resorption and could be induced by excessive body fat mass because adipose tissue is a major source of these cytokines (37, 38). Another possible mechanism is that obesity is often associated with a lower level of adiponectin, an endocrine factor that may cause lower BMD through inhibiting osteoprotegerin expression in osteoblasts (39). Interestingly, the observed negative association between NAFLD markers (USFLI, HSI) and BMD markedly challenges the conventional concept that NAFLD may prevent bone loss because of excessive body weight. In fact, accumulating evidence has suggested that the alterations in the production of several molecular coordinators caused by NAFLD may be detrimental to skeletal health, such as an excessive production of TNF-α (40), and deficiency of osteopontin (41), osteocalcin (42), and osteoprotegerin (43). Additionally, excess intrahepatic lipids could impair insulin sensitivity in NAFLD, which may account for the loss of bone mass (40).

A previous study has shown that with the increased incidence of NAFLD, the prevalence of its related advanced fibrosis increased steadily from 2015–2016 (44). NASH has been reported to accompany by a worse bone condition compared with simple steatosis (12). In the current study, we found that subjects with NAFLD-associated advanced fibrosis have a higher prevalence of fractures compared with controls, although the prevalence of osteopenia/osteoporosis was comparable between the groups. BMD was higher in subjects with NAFLD-related advanced fibrosis and increased with BMI or body weight, whereas the bone strength did not commensurately increase with BMI, and the load-to-strength ratio rose more quickly (45). In addition to bone factors, poorer muscle function caused by NAFLD may also be associated with increased falls and higher risk of fracture (46, 47). Therefore, subjects with NAFLD-related advanced fibrosis may be more likely to fall and be more vulnerable to fracture.

Of significance, our findings suggest that earlier screening and therapeutic intervention for NAFLD could be benefits in improving bone health. It is well-recognized that lifestyle interventions, including diet and exercise, are an effective primary therapy for NAFLD. Reduction in liver fat and improvement of body composition and cardiac function were observed in NAFLD patients who performed appropriate exercise (48). Recent studies have also reported significant beneficial effects on both metabolic and skeletal health by football training in elder individuals with prediabetes (49). Likely, the benefits from exercise on bone status in patients with NAFLD would be highly expected, whereas the evidence is still lacking. Further evidence is also required to determine which form of exercise (resistance, aerobic, or high-intensity intermittent exercise) is suitable for NAFLD patients with poor bone conditions. Additionally, we observed that vitamin D levels appear to be lower in NAFLD patients compared with controls, which is consistent with previous studies (50). At present, the intake of vitamin D from food alone in adults is far from the Dietary Reference Intake (51). Interestingly, Jennings et al. recently reported that the Mediterranean diet, one of the most recommended dietary patterns for NAFLD, together with vitamin D supplements, could prevent bone loss at the femoral neck in patients with osteoporosis (52), suggesting a practicable way to the prevention of osteoporosis for NAFLD patients.

NHANES is a series of elaborately conducted surveys with comprehensive data coverage and consistent data acquisition methods over the period of 2005-2014. Such a set of timely and comprehensive data has ensured the reliability of the current study. However, some limitations exist. First, ultrasonography is most used generally for the diagnosis of NAFLD, which was not available in NHANES surveys. The actual prevalence of NAFLD and its related advanced fibrosis may be underestimated in the U.S. population by using noninvasive panels (USFLI, HSI, and NFS). Second, NHANES did not include longitudinal follow-up data, and thus the nature of our cross-sectional study is unable to explore causality. Third, although the subjects with viral hepatitis, significant consumption of alcohol, and long-term steatogenic medication were excluded from this study, we are unable to exclude other unknown causes, such as primary biliary cholangitis, hemochromatosis, autoimmune hepatitis, and primary sclerosing cholangitis. Fourth, the true prevalence of osteopenia/osteoporosis may be underestimated in the general U.S. population, since noninstitutionalized individuals tend to have higher BMD (53). Fifth, in our analysis, osteoporosis was diagnosed in subject younger than 50 years old owing to the NHANES recommendations about the age categories, which may differ from the ISCD’s (The International Society For Clinical Densitometry) definition of osteoporosis. Finally, although we have adjusted for changes in a variety of demographic and clinical factors in the analysis, the possibility of residual confounders cannot be fully excluded.

In conclusion, the current study reveals the recent downward trend of BMD and the upward trend of osteopenia/osteoporosis in US adults, particularly in individuals with NAFLD, in the period of 2005-2014. It is noted that while the comorbidities of NAFLD including T2D, cardiovascular disease and chronic kidney disease have received considerable attention (54), the problems in bone health have been overlooked in NAFLD patients. Our study suggests more efforts urgently required to fully investigate the complex and interconnected relationship between NAFLD and bone status, and to devise feasible strategies for screening and therapeutic interventions for the individuals with both NAFLD and low BMD.

## Data Availability Statement

The original contributions presented in the study are included in the article/**Supplementary Material**. Further inquiries can be directed to the corresponding author.

## Ethics Statement 

The studies involving human participants were reviewed and approved by National Center for Health Statistics. NHANES obtained written informed consent from all patients/participants, which was approved by the National Center for Health Statistics institutional review board.

## Author Contributions

Concept and design: PX and TZ. Data processing and analyses: TZ, QC, JX, and XJ. Writing of the article: TZ and QC. Review of the manuscript: PX. All authors contributed to the article and approved the submitted version.

## Funding

This work was supported by grants from National Natural Science Foundation of China (81561128014 and 81870559 to PX) and Fudan Distinguished Professorship (to PX).

## Conflict of Interest

The authors declare that the research was conducted in the absence of any commercial or financial relationships that could be construed as a potential conflict of interest.

## Publisher’s Note

All claims expressed in this article are solely those of the authors and do not necessarily represent those of their affiliated organizations, or those of the publisher, the editors and the reviewers. Any product that may be evaluated in this article, or claim that may be made by its manufacturer, is not guaranteed or endorsed by the publisher.

## Supplementary Material

Supplementary Figure 1 Temporal changes in BMD and prevalence of osteopenia/osteoporosis at femoral neck and lumbar spine in participants with or without NAFLD defined by HSI, stratified by age and sex from 2005-2014. BMD is expressed as mean and 95% CI.

## Abbreviations

BMD, bone mineral density; NAFLD, non-alcoholic fatty liver disease; T2DM, type 2 diabetes; NASH, non-alcoholic steatohepatitis; NHANES, National Health and Nutrition Examination Survey; HSI, Hepatic Steatosis Index; USFLI, US Fatty Liver Index; MECs, mobile examination centers; SBP, systolic blood pressure; DBP, diastolic blood pressure; FPG, fasting plasma glucose; HOMA‐IR, Homoeostasis model assessment of insulin resistance; IR, insulin resistance; BMI, Body mass index; MET, metabolic equivalent; CDC, Centers for Disease Control and Prevention; LC-MS/MS, liquid chromatography-tandem mass spectrometric; HSI, Hepatic Steatosis Index; NFS, NAFLD fibrosis score; SE, standard errors; HDL-C, high-density lipoprotein cholesterol; TG, triglyceride; TC, total cholesterol; LDL-C, low-density lipoprotein.
