# Supplementary figures and images for: Prevalence and Trends in Low Bone Density, Osteopenia and Osteoporosis in U.S. Adults With Non-Alcoholic Fatty Liver Disease, 2005–2014
Source: Front Endocrinol (Lausanne). 2022 Jan 19;12:825448. doi: 10.3389/fendo.2021.825448 (PMC8807487; doi:10.3389/fendo.2021.825448)

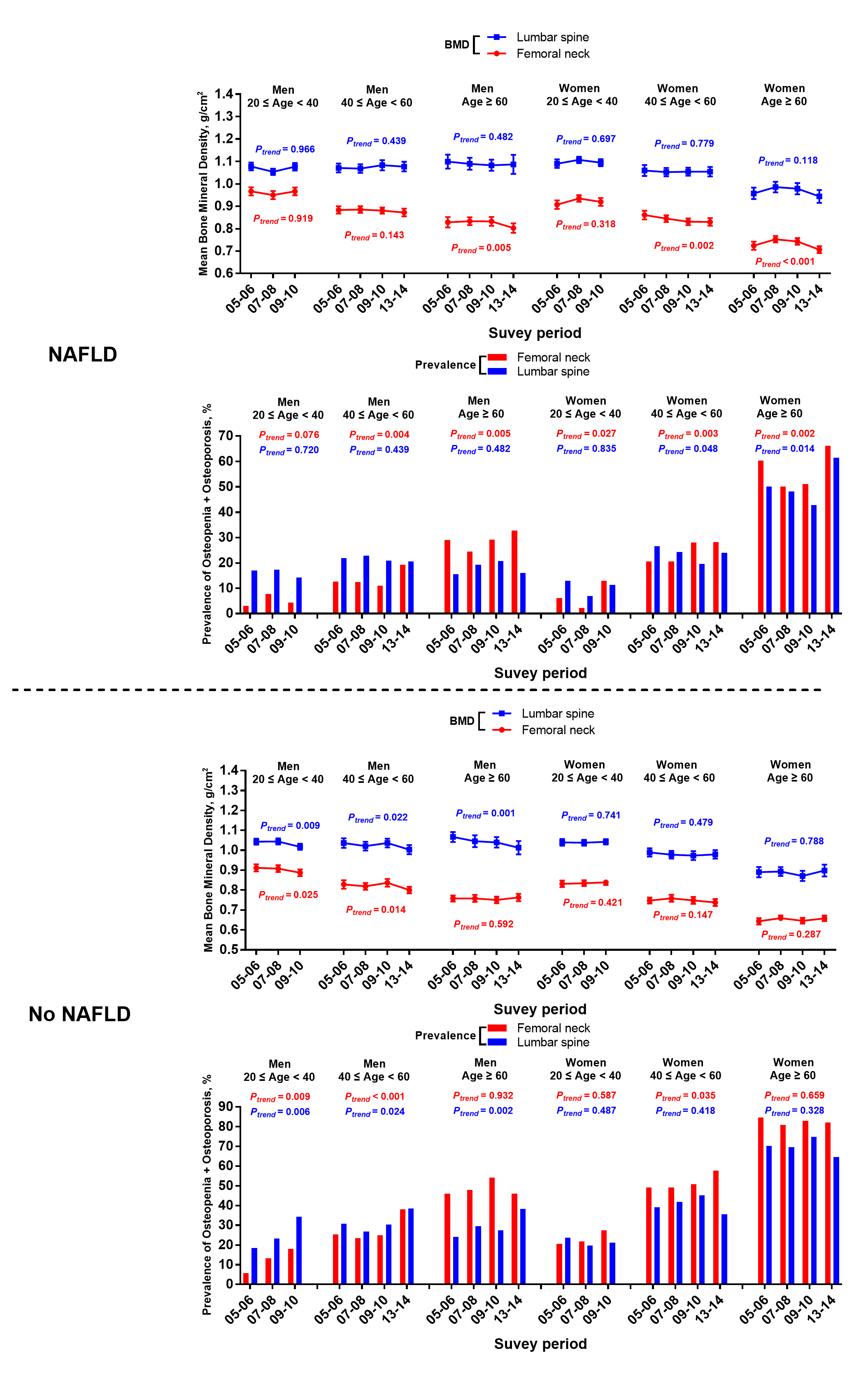

Supplement: Supplementary Figure 1 — Temporal changes in BMD and prevalence of osteopenia/osteoporosis at femoral neck and lumbar spine in participants with or without NAFLD defined by HSI, stratified by age and sex from 2005-2014. BMD is expressed as mean and 95% CI. [file Image_1.tif]
