# Supplementary material for: Prevalence and Trends in Low Bone Density, Osteopenia and Osteoporosis in U.S. Adults With Non-Alcoholic Fatty Liver Disease, 2005–2014
Source: Front Endocrinol (Lausanne). 2022 Jan 19;12:825448. doi: 10.3389/fendo.2021.825448 (PMC8807487; doi:10.3389/fendo.2021.825448)
Supplement: Supplementary file 2 [file DataSheet_1.docx]

**Supporting Information**

**Supplementary Table 1: The equations of the markers (USFLI, HSI, NFS) used in the analysis.**

|  | Full Name | Equation |
| --- | --- | --- |
| USFLI | US Fatty Liver Index | (e^-0.8073*non‐Hispanic black+0.3458*Mexican American+0.0093*age+0.6151* loge (gamma glutamyltransferase [GGT]) +0.0249*waist circumference+1.1792*loge (insulin)+0.8242*loge (glucose) – 14.7812^)/ (1+e^-0.8073*non‐Hispanic black+0.3458*Mexican American+0.0093*age+0.6151* loge (GGT) +0.0249*waist circumference+1.1792*loge (insulin)+0.8242*loge (glucose)– 14.7812^) *100 |
| HSI | Hepatic Steatosis Index | 8*(alanine aminotransferase [ALT]/aspartate aminotransferase [AST] ratio) +BMI (+2, if female; +2, if diabetes mellitus) |
| NFS | NAFLD fibrosis score | [-1.675+0.037*age+0.094*BMI+1.13*impaired fasting glycemia or diabetes (yes= 1, no= 0) +0.99*ALT/AST ratio-0.013*platelet-0.66*albumin] |

**Supplementary Table 2: Characteristics of participants aged ≥ 40 with or without NAFLD defined by HSI through 2005-2014 period.**

|  | No NAFLD | | | | | NAFLD | | | | |
| --- | --- | --- | --- | --- | --- | --- | --- | --- | --- | --- |
|  | **2005-2006** | **2007-2008** | **2009-2010** | **2013-2014** | ***P* for trend** | **2005-2006** | **2007-2008** | **2009-2010** | **2013-2014** | ***P* for trend** |
| NAFLD by HSI |  |  |  |  |  |  |  |  |  |  |
| N | 917 | 1203 | 1181 | 1168 |  | 988 | 1539 | 1652 | 1481 |  |
| Age (years) | 58.11 ± 0.49 | 58.48 ± 0.42 | 58.02 ± 0.42 | 58.40 ± 0.42 | 0.754 | 55.21 ± 0.39 | 55.45 ± 0.33 | 56.20 ± 0.31 | 56.55 ± 0.35 | < 0.001 |
| Male (%) | 46.1 (2.0) | 44.8 (1.8) | 47.7 (1.8) | 50.1 (1.9) | 0.018 | 49.9 (2.0) | 49.2 (1.7) | 48.7 (1.6) | 49.2 (1.7) | 0.750 |
| Race (%) |  |  |  |  | 0.012 |  |  |  |  | 0.341 |
| Mexican American | 4.5 (0.4) | 4.2 (0.4) | 4.3 (0.4) | 4.1 (0.5) |  | 7.3 (0.6) | 6.7 (0.5) | 9.5 (0.6) | 9.2 (0.6) |  |
| Other Hispanic | 2.5 (0.7) | 2.9 (0.3) | 3.4 (0.4) | 4.5 (0.5) |  | 3.1 (0.7) | 5.0 (0.4) | 4.6 (0.4) | 4.5 (0.4) |  |
| Non-Hispanic white | 78.8 (1.4) | 78.0 (1.3) | 77.3 (1.3) | 72.7 (1.3) |  | 75.9 (1.4) | 74.9 (1.2) | 71.9 (1.2) | 70.4 (1.2) |  |
| Non-Hispanic black | 8.3 (0.7) | 7.7 (0.6) | 6.6 (0.6) | 8.2 (0.7) |  | 9.6 (0.7) | 9.1 (0.6) | 10.0 (0.7) | 10.9 (0.7) |  |
| Other races | 5.9 (1.0) | 7.2 (1.1) | 8.4 (1.1) | 10.5 (0.9) |  | 4.1 (0.8) | 4.4 (0.8) | 4.0 (0.7) | 4.9 (0.5) |  |
| BMI (Kg/m^2^) | 24.15 ± 0.11 | 24.16 ± 0.10 | 23.93 ± 0.10 | 24.24 ± 0.11 | 0.526 | 32.01 ± 0.18 | 32.05 ± 0.15 | 32.10 ± 0.14 | 32.56 ± 0.17 | 0.001 |
| Waist circumference (cm) | 88.61 ± 0.41 | 89.31 ± 0.36 | 88.52 ± 0.36 | 90.21 ± 0.39 | < 0.001 | 107.28 ± 0.46 | 107.68 ± 0.38 | 107.37 ± 0.36 | 108.81 ± 0.42 | < 0.001 |
| Current smoker (%) | 22.4 (1.7) | 21.9 (1.5) | 17.2 (1.4) | 17.3 (1.4) | < 0.001 | 17.1 (1.5) | 13.4 (1.1) | 13.0 (1.0) | 13.7 (1.1) | 0.065 |
| Hypertension (%) | 35.4 (1.9) | 37.0 (1.7) | 33.7 (1.6) | 36.9 (1.8) | 0.664 | 46.3 (2.0) | 48.0 (1.7) | 48.3 (1.6) | 48.0 (1.7) | 0.491 |
| Diabetes (%) | 4.8 (0.7) | 5.3 (0.7) | 5.8 (0.7) | 5.3 (0.7) | 0.605 | 14.5 (1.3) | 19.9 (1.3) | 17.7 (1.1) | 21.5 (1.3) | < 0.001 |
| Married status (%) | 70.6 (1.8) | 66.4 (1.7) | 71.2 (1.6) | 70.2 (1.7) | 0.500 | 71.1 (1.7) | 74.9 (1.4) | 71.7 (1.4) | 70.3 (1.5) | 0.136 |
| High education (%) | 83.1 (1.3) | 80.4 (1.3) | 81.8 (1.2) | 86.1 (1.1) | 0.008 | 82.6 (1.3) | 82.0 (1.1) | 80.0 (1.1) | 83.6 (1.1) | 0.367 |
| Poverty (%) | 8.6 (1.0) | 10.2 (0.9) | 9.0 (0.8) | 10.9 (1.0) | 0.126 | 7.4 (0.8) | 8.6 (0.8) | 9.5 (0.7) | 10.9 (0.9) | 0.002 |
| HOMA-IR | 1.80 ± 0.11 | 2.07 ± 0.09 | 2.19 ± 0.08 | 2.12 ± 0.32 | 0.160 | 4.07 ± 0.21 | 5.00 ± 0.28 | 4.93 ± 0.20 | 5.45 ± 0.50 | 0.006 |
| 25(OH)D (nmol/L) | 63.85 ± 0.82 | 73.41 ± 0.92 | 76.16 ± 1.01 | 79.50 ± 1.10 | < 0.001 | 57.65 ± 0.76 | 65.45 ± 0.79 | 67.13 ± 0.77 | 70.49 ± 0.93 | < 0.001 |
| Total cholesterol (mg/dL) | 201.14 ± 1.44 | 203.29 ± 1.41 | 202.12 ± 1.48 | 193.18 ± 1.42 | < 0.001 | 207.62 ± 1.74 | 202.91± 1.44 | 203.13 ± 1.37 | 194.67 ± 1.51 | < 0.001 |
| Triglyceride (mg/dL) | 117.24 ± 4.17 | 118.04 ± 3.74 | 112.47 ± 4.75 | 97.90 ± 3.20 | < 0.001 | 175.26 ± 8.12 | 157.11 ± 4.54 | 148.59 ± 4.35 | 141.93 ± 4.19 | < 0.001 |
| HDL-cholesterol (mg/dL) | 59.20 ± 0.66 | 57.58 ± 0.62 | 59.61 ± 0.65 | 59.12 ± 0.70 | 0.426 | 50.01 ± 0.56 | 48.06 ± 0.42 | 49.68 ± 0.46 | 49.07 ± 0.44 | 0.652 |
| LDL-cholesterol (mg/dL) | 119.58 ± 1.88 | 121.14 ± 1.71 | 118.82 ± 1.81 | 114.57 ± 1.84 | 0.003 | 123.68 ± 2.28 | 120.26 ± 1.76 | 120.86 ± 1.64 | 117.04 ± 1.74 | 0.002 |
| MET (min/week) | - | 4437 ± 242 | 3455 ± 179 | 3496 ± 205 | 0.001 | - | 4583 ± 258 | 4066 ± 219 | 3645 ± 184 | < 0.001 |
| Menopause (%) | 55.4 (2.9) | 64.6 (2.5) | 58.4 (2.6) | 63.5 (2.6) | 0.066 | 66.2 (2.7) | 63.8 (2.4) | 65.1 (2.1) | 70.6 (2.0) | 0.015 |
| Milk intake (%) |  |  |  |  | 0.002 |  |  |  |  | < 0.001 |
| Never | 15.5 (1.5) | 13.6 (1.4) | 17.6 (1.4) | 19.2 (1.4) |  | 15.1 (1.5) | 16.2 (1.2) | 16.9 (1.2) | 18.4 (1.3) |  |
| Rarely | 13.6 (1.4) | 12.6 (1.3) | 12.4 (1.2) | 15.1 (1.4) |  | 15.4 (1.4) | 13.4 (1.2) | 13.3 (1.1) | 16.2 (1.2) |  |
| Sometimes | 21.0 (1.6) | 26.6 (1.7) | 23.9 (1.6) | 23.3 (1.6) |  | 28.8 (1.8) | 29.6 (1.6) | 28.6 (1.5) | 30.6 (1.6) |  |
| Often | 49.4 (2.0) | 42.4 (1.8) | 45.9 (1.8) | 42.2 (1.9) |  | 40.2 (1.9) | 40.4 (1.7) | 41.0 (1.6) | 34.6 (1.6) |  |
| Varied | 0.5 (0.3) | 0.6 (0.3) | 0.2 (0.2) | 0.2 (0.1) |  | 0.4 (0.3) | 0.4 (0.2) | 0.1 (0.1) | 0.2 (0.1) |  |
| BMD (g/cm^2^) |  |  |  |  |  |  |  |  |  |  |
| Femoral neck | 0.74 ± 0.005 | 0.75 ± 0.005 | 0.75 ± 0.005 | 0.74 ± 0.005 | 0.313 | 0.84 ± 0.005 | 0.84 ± 0.004 | 0.83 ± 0.004 | 0.81 ± 0.005 | < 0.001 |
| Lumbar spine | 1.00 ± 0.006 | 0.98 ± 0.006 | 0.99 ± 0.007 | 0.98 ± 0.007 | 0.032 | 1.05 ± 0.007 | 1.05 ± 0.006 | 1.06 ± 0.006 | 1.05 ± 0.007 | 0.432 |

Data are expressed as mean ± SE for continuous variables or the proportion (SE) for categorical variables. ^1^MET score calculation was not available in 2005-2006 period. ^2^The proportion of Menopause was calculated in women.

**Supplementary Table 3: Characteristics of participants aged 20-39 with or without NAFLD through 2005-2010 period.**

|  | No NAFLD | | | | NAFLD | | | |
| --- | --- | --- | --- | --- | --- | --- | --- | --- |
|  | **2005-2006** | **2007-2008** | **2009-2010** | ***P* for trend** | **2005-2006** | **2007-2008** | **2009-2010** | ***P* for trend** |
| NAFLD by USFLI |  |  |  |  |  |  |  |  |
| N | 360 | 438 | 467 |  | 78 | 110 | 166 |  |
| Age (years) | 29.71 ± 0.37 | 29.12 ± 0.31 | 28.78 ± 0.31 | 0.024 | 31.29 ± 0.70 | 31.68 ± 0.60 | 30.70 ± 0.57 | 0.339 |
| Male (%) | 49.0 (3.1) | 49.5 (2.9) | 49.2 (2.8) | 0.951 | 62.9 (6.5) | 61.0 (5.6) | 68.3 (4.3) | 0.332 |
| Race (%) |  |  |  | 0.407 |  |  |  | 0.174 |
| Mexican American | 10.4 (1.3) | 9.3 (1.1) | 9.2 (1.0) |  | 23.0 (4.2) | 24.8 (4.0) | 21.4 (3.0) |  |
| Other Hispanic | 6.2 (1.5) | 6.0 (0.9) | 7.5 (1.0) |  | 9.8 (3.8) | 5.9 (1.8) | 8.6 (1.9) |  |
| Non-Hispanic white | 62.8 (2.7) | 62.9 (2.5) | 60.6 (2.5) |  | 61.6 (5.8) | 59.8 (5.2) | 55.6 (4.6) |  |
| Non-Hispanic black | 14.0 (1.5) | 12.2 (1.3) | 13.9 (1.5) |  | 4.9 (1.9) | 4.0 (1.5) | 8.2 (2.0) |  |
| Other races | 6.5 (1.6) | 9.5 (1.8) | 8.8 (1.8) |  | 0.8 (0.8) | 5.4 (2.8) | 6.2 (2.6) |  |
| BMI (Kg/m^2^) | 25.53 ± 0.28 | 25.54 ± 0.27 | 25.58 ± 0.25 | 0.877 | 33.49 ± 0.49 | 33.51 ± 0.57 | 33.25 ± 0.45 | 0.105 |
| Waist circumference (cm) | 88.10 ± 0.69 | 87.84 ± 0.69 | 87.93 ± 0.64 | 0.836 | 109.35 ± 1.10 | 110.19 ± 1.39 | 109.16 ± 0.99 | 0.817 |
| Current smoker (%) | 23.1 (2.7) | 24.3 (2.4) | 26.0 (2.5) | 0.343 | 22.6 (5.3) | 21.7 (4.3) | 22.4 (4.1) | 0.982 |
| Hypertension (%) | 3.7 (1.1) | 5.5 (1.4) | 3.4 (1.1) | 0.816 | 14.7 (4.7) | 13.4 (3.5) | 12.4 (3.3) | 0.604 |
| Diabetes (%) | 1.6 (0.9) | 1.3 (0.6) | 1.2 (0.6) | 0.697 | 7.0 (3.4) | 8.7 (2.7) | 7.5 (2.2) | 0.927 |
| Married status (%) | 62.8 (3.0) | 57.7 (2.8) | 51.2 (2.8) | 0.001 | 73.0 (5.8) | 64.6 (5.6) | 62.5 (4.7) | 0.105 |
| High education (%) | 87.4 (1.8) | 84.9 (1.8) | 85.3 (1.6) | 0.386 | 81.0 (4.4) | 77.0 (4.4) | 77.7 (3.5) | 0.590 |
| Poverty (%) | 10.8 (1.6) | 13.3 (1.6) | 18.2 (2.0) | 0.003 | 12.1 (3.5) | 25.9 (4.7) | 25.9 (4.2) | 0.022 |
| HOMA-IR | 1.72 ± 0.06 | 1.98 ± 0.05 | 2.12 ± 0.05 | < 0.001 | 5.40 ± 0.29 | 6.29 ± 0.45 | 6.49 ± 0.37 | 0.055 |
| 25(OH)D (nmol/L) | 62.36 ± 1.39 | 68.62 ± 1.78 | 65.23 ± 1.36 | 0.122 | 58.81 ± 2.47 | 57.33 ± 2.42 | 54.84 ± 1.65 | 0.099 |
| Blood cadmium (ug/L) | 0.41 ± 0.02 | 0.43 ± 0.02 | 0.47 ± 0.03 | 0.105 | 0.36 ± 0.04 | 0.36 ± 0.03 | 0.38 ± 0.05 | 0.736 |
| Total cholesterol (mg/dL) | 184.08 ± 2.24 | 179.68 ± 2.00 | 180.23 ± 1.82 | 0.123 | 200.34 ± 4.89 | 205.80 ± 4.61 | 197.27 ± 3.82 | 0.426 |
| Triglyceride (mg/dL) | 106.21 ± 4.50 | 102.31 ± 3.97 | 101.72 ± 3.30 | 0.410 | 193.46 ± 15.43 | 198.04 ± 12.39 | 171.52 ± 12.68 | 0.222 |
| HDL-cholesterol (mg/dL) | 55.59 ± 0.87 | 53.02 ± 0.79 | 54.10 ± 0.79 | 0.140 | 45.08 ± 1.47 | 41.51 ± 1.10 | 42.40 ± 0.83 | 0.085 |
| LDL-cholesterol (mg/dL) | 107.52 ± 2.05 | 106.58 ± 1.85 | 105.87 ± 1.65 | 0.460 | 116.95 ± 4.26 | 127.59 ± 4.04 | 124.03 ± 3.47 | 0.201 |
| MET (min/week) | - | 6432 ± 469 | 5125 ± 395 | 0.016 | - | 8361 ± 1284 | 4379 ± 476 | < 0.001 |
| Milk intake (%) |  |  |  | 0.220 |  |  |  | 0.207 |
| Never | 12.8 (2.0) | 12.9 (1.8) | 13.9 (2.0) |  | 9.6 (3.5) | 17.0 (4.5) | 10.1 (2.9) |  |
| Rarely | 13.8 (2.1) | 12.7 (1.8) | 12.5 (1.7) |  | 21.9 (5.3) | 18.8 (4.4) | 14.4 (3.2) |  |
| Sometimes | 26.3 (2.7) | 32.0 (2.7) | 34.4 (2.6) |  | 34.5 (6.4) | 26.3 (4.7) | 32.7 (4.5) |  |
| Often | 46.9 (3.1) | 41.8 (2.8) | 39.2 (2.7) |  | 34.0 (6.3) | 35.9 (5.6) | 42.7 (4.8) |  |
| Varied | 0.2 (0.2) | 0.5 (0.4) | 0.0 (0.0) |  | 0.0 (0.0) | 2.0 (2.0) | 0.0 (0.0) |  |
| BMD (g/cm^2^) |  |  |  |  |  |  |  |  |
| Femoral neck | 0.89 ± 0.008 | 0.90 ± 0.008 | 0.88 ± 0.008 | 0.408 | 0.97 ± 0.015 | 0.96 ± 0.012 | 0.95 ± 0.014 | 0.346 |
| Lumbar spine | 1.05 ± 0.007 | 1.06 ± 0.007 | 1.04 ± 0.007 | 0.221 | 1.08 ± 0.014 | 1.06 ± 0.012 | 1.06 ± 0.010 | 0.121 |
| NAFLD by HSI |  |  |  |  |  |  |  |  |
| N | 525 | 664 | 733 |  | 481 | 616 | 689 |  |
| BMD (g/cm^2^) |  |  |  |  |  |  |  |  |
| Femoral neck | 0.87 ± 0.006 | 0.87 ± 0.006 | 0.86 ± 0.005 | 0.243 | 0.94 ± 0.007 | 0.94 ± 0.006 | 0.95 ± 0.006 | 0.477 |
| Lumbar spine | 1.04 ± 0.006 | 1.04 ± 0.005 | 1.03 ± 0.005 | 0.107 | 1.08 ± 0.007 | 1.08 ± 0.005 | 1.09 ± 0.007 | 0.850 |

Data are expressed as mean ± SE for continuous variables or the proportion (SE) for categorical variables. ^1^MET score calculation was not available in 2005-2006 period.

**Supplementary Table 4: Trends in prevalence of osteopenia/osteoporosis in participants aged ≥ 40 with or without NAFLD defined by HSI through 2005-2014 period.**

|  | No NAFLD | | | | | NAFLD | | | | |
| --- | --- | --- | --- | --- | --- | --- | --- | --- | --- | --- |
|  | **2005-2006** | **2007-2008** | **2009-2010** | **2013-2014** | ***P* for trend** | **2005-2006** | **2007-2008** | **2009-2010** | **2013-2014** | ***P* for trend** |
| NAFLD by HSI |  |  |  |  |  |  |  |  |  |  |
| Femoral neck |  |  |  |  |  |  |  |  |  |  |
| Prevalence (%) | 49.9 | 49.6 | 50.9 | 54.8 |  | 25.7 | 23.7 | 27.2 | 34.3 |  |
| Model 1 | 1.00 (Ref.) | 0.89 (0.69-1.15) | 1.03 (0.80-1.33) | 1.34 (1.02-1.74) | 0.020 | 1.00 (Ref.) | 0.87 (0.66-1.13) | 0.98 (0.76-1.27) | 1.55 (1.19-2.02) | < 0.001 |
| Model 2 | 1.00 (Ref.) | 0.93 (0.71-1.22) | 1.07 (0.82-1.38) | 1.40 (1.06-1.84) | 0.021 | 1.00 (Ref.) | 0.94 (0.71-1.23) | 1.04 (0.79-1.35) | 1.69 (1.29-2.22) | < 0.001 |
| Model 3 | 1.00 (Ref.) | 0.88 (0.59-1.32) | 1.04 (0.71-1.53) | 1.56 (1.05-2.33) | 0.024 | 1.00 (Ref.) | 1.04 (0.69-1.57) | 1.24 (0.83-1.83) | 2.01 (1.35-2.99) | 0.001 |
| Model 4 | 1.00 (Ref.) | 0.86 (0.57-1.31) | 1.06 (0.73-1.55) | 1.53 (1.03-2.29) | 0.027 | 1.00 (Ref.) | 1.05 (0.70-1.59) | 1.28 (0.86-1.90) | 2.03 (1.36-3.02) | 0.001 |
| Model 5 | - | 1.00 (Ref.) | 1.27 (0.81-1.98) | 1.79 (1.14-2.83) | 0.036 | - | 1.00 (Ref.) | 1.15 (0.71-1.88) | 2.09 (1.31-3.34) | 0.003 |
| Lumbar spine |  |  |  |  |  |  |  |  |  |  |
| Prevalence (%) | 40.0 | 40.9 | 42.5 | 41.6 |  | 26.9 | 26.7 | 24.0 | 28.0 |  |
| Model 1 | 1.00 (Ref.) | 1.04 (0.79-1.36) | 1.14 (0.85-1.52) | 1.14 (0.85-1.53) | 0.747 | 1.00 (Ref.) | 0.97 (0.73-1.28) | 0.81 (0.61-1.08) | 1.14 (0.85-1.52) | 0.128 |
| Model 2 | 1.00 (Ref.) | 1.02 (0.77-1.35) | 1.14 (0.85-1.52) | 1.15 (0.85-1.56) | 0.714 | 1.00 (Ref.) | 1.00 (0.75-1.32) | 0.83 (0.62-1.11) | 1.17 (0.87-1.57) | 0.128 |
| Model 3 | 1.00 (Ref.) | 1.15 (0.76-1.76) | 1.08 (0.70-1.68) | 1.53 (0.99-2.39) | 0.236 | 1.00 (Ref.) | 1.45 (0.96-2.20) | 1.15 (0.75-1.77) | 1.73 (1.12-2.66) | 0.063 |
| Model 4 | 1.00 (Ref.) | 1.13 (0.73-1.74) | 1.09 (0.70-1.69) | 1.53 (0.97-2.36) | 0.271 | 1.00 (Ref.) | 1.45 (0.95-2.21) | 1.21 (0.79-1.86) | 1.73 (1.12-2.69) | 0.082 |
| Model 5 | - | 1.00 (Ref.) | 1.04 (0.63-1.70) | 1.51 (0.91-2.50) | 0.193 | - | 1.00 (Ref.) | 0.80 (0.49-1.32) | 1.22 (0.74-2.01) | 0.275 |

Data are expressed as OR (95% CI). Model 1 was adjusted for sex, age, race, BMI, waist circumference smoking, educational, marital, and economic status. Model 2 was adjusted for the adjustments of model 1 plus nutritional status, 25(OH)D and milk intake included. Model 3 was adjusted for hypertension, diabetes, HDL-C, TG, TC, and LDL-C in addition to model 2. Model 4 was adjusted for the adjustments of model 3 plus menopausal status. Model 5 was further adjusted for physical activity, not available for 2005-2006 period.

**Supplementary Table 5: Bone mineral density in participants with or without NAFLD.**

|  | 20 ≤ Age < 40 | | | Age ≥ 40 | | | | Age ≥ 40 | | |
| --- | --- | --- | --- | --- | --- | --- | --- | --- | --- | --- |
|  | **No NAFLD** | **NAFLD** | ***P*** | **No NAFLD** | **NAFLD** | ***P*** | **No NAFLD or Advanced Fibrosis** | | **NAFLD with Advanced Fibrosis** | ***P*** |
| NAFLD by USFLI |  |  |  |  |  |  |  | |  |  |
| Femoral neck BMD | 0.89 ± 0.005 | 0.96 ± 0.008 | < 0.001 | 0.78 ± 0.003 | 0.83 ± 0.005 | < 0.001 | 0.80 ± 0.003 | | 0.83 ± 0.018 | < 0.001 |
| Lumbar spine BMD | 1.05 ± 0.004 | 1.07 ± 0.007 | < 0.001 | 1.01 ± 0.004 | 1.05 ± 0.006 | < 0.001 | 1.02 ± 0.003 | | 1.06 ± 0.025 | < 0.001 |
| NAFLD by HSI |  |  |  |  |  |  |  | |  |  |
| Femoral neck BMD | 0.87 ± 0.003 | 0.94 ± 0.004 | < 0.001 | 0.75 ± 0.002 | 0.83 ± 0.002 | < 0.001 | 0.79 ± 0.002 | | 0.82 ± 0.011 | < 0.001 |
| Lumbar spine BMD | 1.04 ± 0.003 | 1.08 ± 0.003 | < 0.001 | 0.99 ± 0.003 | 1.05 ± 0.003 | < 0.001 | 1.02 ± 0.002 | | 1.09 ± 0.018 | < 0.001 |

Data are expressed as mean ± SE.

**Supplementary Table 6: The association between NAFLD (defined by HSI) and NAFLD related advanced fibrosis and odds of fracture in participants aged ≥ 40.**

| NAFLD by HSI | No NAFLD | NAFLD | *P* | No NAFLD or Advanced Fibrosis | NAFLD with Advanced Fibrosis | *P* |
| --- | --- | --- | --- | --- | --- | --- |
| Hip fracture |  |  |  |  |  |  |
| Prevalence (%) | 1.6 | 1.2 |  | 1.4 | 1.2 |  |
| Model 1 | 1.00 (Ref.) | 0.75 (0.41-1.37) | 0.348 | 1.00 (Ref.) | 0.73 (0.25-2.14) | 0.568 |
| Model 2 | 1.00 (Ref.) | 0.74 (0.41-1.35) | 0.328 | 1.00 (Ref.) | 0.72 (0.25-2.12) | 0.551 |
| Model 3 | 1.00 (Ref.) | 1.20 (0.48-3.02) | 0.696 | 1.00 (Ref.) | 0.79 (0.19-3.31) | 0.743 |
| Model 4 | 1.00 (Ref.) | 1.16 (0.47-2.88) | 0.744 | 1.00 (Ref.) | 0.86 (0.21-3.54) | 0.828 |
| Model 5 | 1.00 (Ref.) | 1.78 (0.35-8.94) | 0.484 | 1.00 (Ref.) | 2.84 (0.39-20.91) | 0.305 |
| Spine fracture |  |  |  |  |  |  |
| Prevalence (%) | 2.2 | 2.5 |  | 2.3 | 3.1 |  |
| Model 1 | 1.00 (Ref.) | 0.98 (0.59-1.61) | 0.930 | 1.00 (Ref.) | 1.42 (0.62-3.29) | 0.411 |
| Model 2 | 1.00 (Ref.) | 1.03 (0.62-1.71) | 0.915 | 1.00 (Ref.) | 1.40 (0.61-3.26) | 0.430 |
| Model 3 | 1.00 (Ref.) | 1.38 (0.66-2.90) | 0.386 | 1.00 (Ref.) | 1.49 (0.48-4.55) | 0.489 |
| Model 4 | 1.00 (Ref.) | 1.39 (0.67-2.89) | 0.372 | 1.00 (Ref.) | 1.48 (0.48-4.55) | 0.492 |
| Model 5 | 1.00 (Ref.) | 2.29 (0.89-5.89) | 0.087 | 1.00 (Ref.) | 4.05 (1.16-14.14) | 0.028 |

Data are expressed as OR (95% CI). Model 1 was adjusted for sex, age, race, BMI, waist circumference smoking, educational, marital, and economic status. Model 2 was adjusted for the adjustments of model 1 plus nutritional status, 25(OH)D and milk intake included. Model 3 was adjusted for hypertension, diabetes, HDL-C, TG, TC, and LDL-C in addition to model 2. Model 4 was adjusted for the adjustments of model 3 plus menopausal status. Model 5 was further adjusted for physical activity, not available for 2005-2006 period.
